# Combinatorial Effects of Double Cardiomyopathy Mutant Alleles in Rodent Myocytes: A Predictive Cellular Model of Myofilament Dysregulation in Disease

**DOI:** 10.1371/journal.pone.0009140

**Published:** 2010-02-10

**Authors:** Jennifer Davis, Joseph M. Metzger

**Affiliations:** Department of Integrative Biology and Physiology, University of Minnesota, Minneapolis, Minnesota, United States of America; Istituto Dermopatico dell'Immacolata, Italy

## Abstract

Inherited cardiomyopathy (CM) represents a diverse group of cardiac muscle diseases that present with a broad spectrum of symptoms ranging from benign to highly malignant. Contributing to this genetic complexity and clinical heterogeneity is the emergence of a cohort of patients that are double or compound heterozygotes who have inherited two different CM mutant alleles in the same or different sarcomeric gene. These patients typically have early disease onset with worse clinical outcomes. Little experimental attention has been directed towards elucidating the physiologic basis of double CM mutations at the cellular-molecular level. Here, dual gene transfer to isolated adult rat cardiac myocytes was used to determine the primary effects of co-expressing two different CM-linked mutant proteins on intact cardiac myocyte contractile physiology. Dual expression of two CM mutants, that alone moderately increase myofilament activation, tropomyosin mutant A63V and cardiac troponin mutant R146G, were shown to additively slow myocyte relaxation beyond either mutant studied in isolation. These results were qualitatively similar to a combination of moderate and strong activating CM mutant alleles αTmA63V and cTnI R193H, which approached a functional threshold. Interestingly, a combination of a CM myofilament deactivating mutant, troponin C G159D, together with an activating mutant, cTnIR193H, produced a hybrid phenotype that blunted the strong activating phenotype of cTnIR193H alone. This is evidence of neutralizing effects of activating/deactivating mutant alleles in combination. Taken together, this combinatorial mutant allele functional analysis lends molecular insight into disease severity and forms the foundation for a predictive model to deconstruct the myriad of possible CM double mutations in presenting patients.

## Introduction

Inherited cardiomyopathies (CM) are the most prevalent genetic disorders of the cardiovascular system [1]. They typically have an autosomal dominant mode of inheritance and comprise a spectrum of clinically heterogeneous phenotypes [1]. Cardiomyopathies have been categorized into three clinical subtypes, hypertrophic (HCM), dilated (DCM) and restrictive (RCM) cardiomyopathy, based on both morphologic and functional criteria [2], [3]. Given the complexity of this disease, inherited cardiomyopathy is now broadly defined by poor myocardial performance including diastolic dysfunction, a high risk for sudden cardiac death and histopathology [3]. Despite the varied natural histories of the inherited CM subtypes, the genetic basis of these diseases converge on one subcellular structure, the cardiac sarcomere [4]. The sarcomere is the primary functional unit of cardiac muscle that is responsible for contraction and force generation. Hundreds of mutations in eleven different sarcomeric genes have co-segregated with a cardiomyopathy phenotype [5]. In genotyped patients, the CM prognosis and penetrance can be highly variable between identified mutations in the same or different gene as well as within kindreds [6], [7].

Some of the genetic complexity associated with CM can be attributed to environmental factors and genetic modifiers. However, emerging complexity in the form of multiple CM allele inheritance is also evident [8]–[14]. Due to the high prevalence of CM alleles [5], [13] it is not surprising that individuals with compound heterozygous (different CM mutate alleles within the same gene) or double heterozygous (CM mutations in two different alleles) genotypes have been documented and likely this population contributes to the overall clinical heterogeneity [9]. Approximately, 5% of CM patients are double heterozygous [9], and β-myosin heavy chain (β-MHC) and myosin binding protein-C (MyBP-C) are generally the affected genetic loci in these individuals [8], [10]–[18]. These loci logically predominate as the highest percentage of CM cases have single mutations in these two genes [10], [17]. There are, however, documented cases of compound homozygous cardiac troponin T (cTnT) patients [18]–[20] as well as double heterozygous patients with mutations in MyBP-C + cardiac troponin T (cTnT), MyBP-C + tropomyosin (αTm), MyBP-C + cardiac troponin I (cTnI), and β-MHC + cTnT [9], [11]–[15], [17], [20], [21]. Disease in these kindreds tends to be more severe, with CM rapidly progressing to greater left ventricular hypertrophy, loss of cardiac function, and increased incidence of sudden cardiac death [9]–[12], [14]–[18].

One hypothesis explaining these findings is that a higher total dosage of CM mutant protein is causal for the increased disease severity [9]. This hypothesis finds support from both animal and *in vitro* gene-dosage experiments [9], [22]–[25]. Homozygous and double heterozygous transgenic mouse models of HCM have recapitulated increased disease severity and loss of cardiac function similar to phenotypes observed in humans [12], [18], [23], [24], [26]–[28]. While these data are supportive of the dose-response hypothesis, the molecular underpinnings of this heightened disease severity on myocyte function remain largely unanswered. In addition, protein dosage has not yet been determined in compound allele CM patients lending some uncertainty to this dosage hypothesis in humans.

An alternative hypothesis explored here is that multiple CM mutant proteins cause allele-specific combinatorial primary effects that uniquely alter sarcomeric function and are not necessarily linked directly to total mutant gene dosage. Thus, our working hypothesis is that altered biophysical properties of double mutant sarcomeric proteins could interact to either heighten *or* mitigate the functional cellular outcomes of either mutant allele when studied in isolation. A prediction of this model is that individuals inheriting two mutant CM alleles with opposing biophysical properties may paradoxically have a less severe outcome than either parent with only a single CM mutant allele. Adding to the complexity of CM is the realization there are ∼467 CM-linked mutant alleles that have been reported in eleven different sarcomeric genes [5]. Excluding homozygous genotypes this translates to 102,000 possible compound/double mutant allele combinations ([(467^2^)−467)/2]), with ∼75,000 (102,000-compound heterozygous combinations) of those totaling the number of double heterozygous genotypes. The total number of compound heterozygous combinations was derived by the following formula: ((X^2^)−X)/2, where X is the number of mutations identified for a given sarcomeric gene. This formula is calculated for total number of mutations within each sarcomeric loci and then summated to equal 33,737 possible genotypes.

In light of the staggering number of potential compound/double genotypes, an examination of every possible double mutant combination is unfeasible. We reasoned that a predictive model, predicated on experimental data, could be implemented as an index of functional outcomes in multiple CM allele inheritance. To facilitate this undertaking we assumed that specific CM mutants could be categorized as Ca^2+^ activating (thin filament activator) or Ca^2+^deactivating (thin filament deactivator) archetypes. Results derived from these archetypes could then inform a predictive model for extrapolation to all possible CM mutant combinations. The study employed CM mutant thin filament regulatory proteins as a proof-of-concept methodology to address this problem for three reasons. First, this family of sarcomeric proteins is highly amenable to acute genetic engineering and structure-function studies [22], [29]–[34]. Second, there is a wealth of previous data on effects of single thin filament CM mutations on cardiac myocyte physiology [29], [35]–[37]. Third, while this data is focused on CM mutations in the thin filament regulatory system, nearly all CM mutants including the more prevalent thick filament mutants impact myofilament activation, either positively or negatively, to directly alter myofilament Ca^2+^ sensitivity and thin filament cooperativity thereby modifying myocyte force development and relaxation performance [38]–[41]. Thick filament proteins myosin and MyBP-C also affect myocyte function via established reciprocal interactions between myosin cross-bridge cycling and thin filament activation [42]. Thus, myosin and MyBP-C CM mutants converge to affect the activation properties of the thin filament and can be analyzed by this activation paradigm. Given the central role of thin filament regulation on myocyte performance and the realization that both thin and thick filament CM mutant proteins directly alter myofilament activation [39], [42]–[44], we reasoned that representative thin filament CM mutants could serve as surrogates for the breadth of thin and thick filament CM mutant alleles.

**Figure 1:**
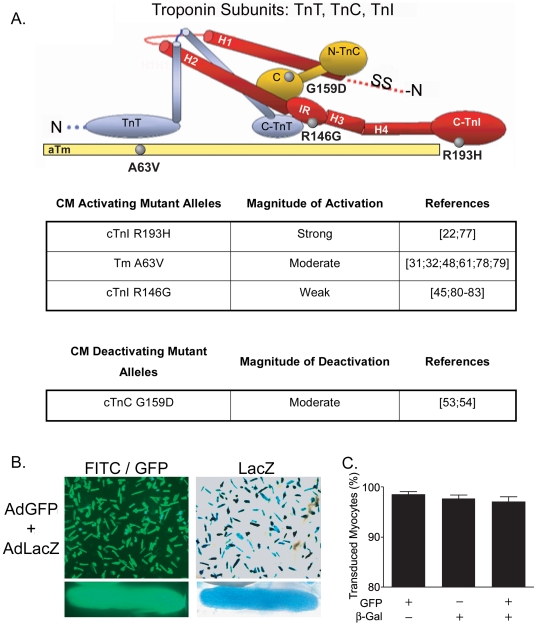
Thin filament regulatory protein targets and efficiency of dual gene transfer. (**A**) Schematic representation of the troponin subunits, troponin I (TnI), troponin T (TnT), and troponin C (TnC) and tropomyosin (Tm) the location of CM-linked mutations (gray circles) with in the secondary structure of troponin and tropomyosin. (**B**) Representative images (10X) of cultured adult rat cardiac myocytes co-transduced with the eGFP (200 MOI) and Lac Z genes (200 MOI) 3 days post gene transfer. Bottom panel is a 40X image of one myocyte expressing both eGFP and β-galactosidase. (**C**) Summary of the ratio of transduced to total myocytes in a field. Approximately 98% of myocytes are expressing both eGFP and β-galactosidase by day four in cultured myocytes co-transduced with adenovirus containing the eGFP and Lac Z genes. Data is presented as mean+SEM, n = 5 preparations.

To derive a working model predicting the physiologic effects of multiple CM alleles, experiments combined CM alleles that separately cause mild, moderate or strong myofilament activation (Figure 1A). A combination of a myofilament activator together with a myofilament deactivator was also tested in separate experiments. Findings show that two CM mutant myofilament activator alleles together have additive effects to further activate the myofilament, and manifest functionally as worsened relaxation performance. Activator alleles also show evidence of threshold effects on cellular performance suggesting there exists saturation points for myofilament dysregulation. The combination of an activating CM mutant with a deactivating CM mutant caused a hybrid phenotype that neutralized the primary defects caused by these CM mutants in isolation. Together these data provide the basis for a predictive model offering new insight into the functional ramifications of multiple CM mutant allele inheritance.

## Methods

### Generation of Recombinant Adenovirus

Adenoviral construction of AdGFP, AdLacZ, αTmE180G, αTmK70T, αTmA63V, cTnIR146G, and cTnIR193H were previously described [22], [31], [45]. All cTnI constructs were derived from the full-length rodent sequence and tagged with C-terminal Flag epitope (DYKDDDDK, Sigma) to distinguish the recombinant from the native cTnI; while, the Tm mutants were engineered into the full-length human Tm [22], [31], [45]. Stratagene Quik Change site directed mutagenesis kit was used to engineer the G159D into full-length human cTnC. All mutants were confirmed by sequencing. G159D cTnC was subcloned into the pDC315 AdMax shuttle vector (Microbix Biosystems Inc.) with and without an HA (Hemagglutinin) epitope tag as previously described [22]. For all functional studies wild type (WT) represents the control group which consisted of combined data from non-transduced, AdcTnIwildtype, and AdcTnIFlag, as we have shown previously that the expression of the thin filament proteins with and without a flag tag are functionally innocuous [22], [31], [45]. Viral particle lysates were prepared for infection of a cell factory and plaque assays were used for determining the viral titer in plaque forming units/ml (pfu/ml) as previously described. The average titer for viral stocks ranged from 10^10^–10^12^ pfu/ml.

### Adult Rat Cardiac Myocyte Isolation and Dual Adenoviral Gene Transfer

Rat hearts were excised from heparinized (1500 U/kg) and anesthetized (Nembutal; 150 U/kg) adult female Sprague Dawley rats (200 g, Harlan, Indianapolis, IN). Ventricular cardiac myocytes were isolated via collagenase-hyaluronidase digestion as previously described [33]. The average yield from rat myocyte isolations is 1.8×10^6^ cells with 77% viability. The care and use of the laboratory animals for this study was in agreement with the guidelines set forth by the Internal Review Board of the University of Michigan Committee on the Use and Care of Animals. Veterinary care was provided by the University of Michigan Unit for Laboratory Animal Medicine.

Post digestion the myocytes were resuspended in DMEM (Dulbecco's Minimum Eagle Media) plus 5% fetal bovine serum (FBS) and approximately 20,000 cells were plated on laminin-coated coverslips and incubated for 2 hours. At which time the media is replaced with serum-free DMEM. Subsequently myocytes are transduced with recombinant adenoviral vectors. For single gene transfer myocytes were transduced with an experimentally predetermine optimal multiplicity of infection (MOI, range from 20–500) based on the stoichiometric replacement for each vector and level of efficiency. For dual gene transfer two different recombinant adenoviruses were combined so that the total MOI was 500 and then simultaneously applied to the plated myocytes [46].

### Protein Expression and Western Blot Analysis

To assess the mutant cTnIs expression and incorporation into the sarcomere, cultured ventricular myocytes were scraped into Laemmli sample buffer and prepared for gel electrophoresis by boiling and sonication. As previously described, cardiac proteins were separated on a 4–20% gradient SDS-page gel (Criterion, Bio Rad) and prepared for Western blot analysis. A cTnI specific polyclonal antibody (AB1927, 1∶1000, Chemicon) and Tm specific monoclonal antibody (Tm-311, Sigma, 1∶10^6^) were used for immunodetection. In some instances horseradish peroxidase-conjugated goat anti-mouse or goat anti-rabbit secondary antibodies were used for chemilumenescence detection (1∶2000, Amersham) [45], [47], while other assays used fluorophore conjugated goat anti-rabbit and goat anti-mouse secondary antibodies (1∶5000, Molecular Probes, Invitrogen) for detection by Odyssey (Licor Biosciences).

### Unloaded Dynamic Sarcomere Contractility and Ca^2+^ Transients

Sarcomere length measurements and Ca^2+^ transients were measured simultaneously using the Ionoptix system (Ionoptix Co.) as previously described. A coverslip containing cultured myocytes was bathed in HEPES buffered medium 199 (M199, 1.8 mM [Ca^2+^]) kept at 37°C. Myocytes were electrically paced at 40 volts with a frequency of 0.2 Hz. Ca^2+^ transient measurements were accomplished using the excitable Ca^2+^ indicator [22], [46], Fura-2 AM (Molecular Probes, Invitrogen). Cultured cardiac myocytes were loaded with 5 µM Fura-2 for 5 minutes followed by a 15 minute deesterification period as previously described [22], [46]. Functional measurements were made on myocytes (5–15 per preparation) obtained from a minimum of 3 preparations.

## Results

### Efficiency and Targeted Stoichiometric Replacement from Thin Filament Dual Gene Transfer

To validate the experimental design of dual mutant CM gene transfer, we determined the efficiency of adenoviral-based dual gene transfer using reporter genes (Figure 1). Acutely isolated adult rat cardiac myocytes were simultaneously transduced with two reporter viral vectors AdeGFP and AdLacZ. Efficiency of transduction was tracked across time in primary culture. Figure 1B displays representative images of myocytes after dual gene transfer, demonstrating the high transduction efficiency of this approach. After dual gene transfer, 98% of myocytes co-expressed eGFP and β-galactosidase (Figure 1C) comparable to previous reports of adenoviral mediated dual gene transfer [48]. Dual gene transfer transduction efficiency was not different when compared to the efficiency of transduction with a single reporter gene construct (Figure 1C) [49]. These data show that dual gene transfer is as rapid and highly efficient as previous reports of single adenoviral transduction [22], [31], [34], [45].

### Dual Expression of CM Mutant Myofilament Activators

**Figure 2:**
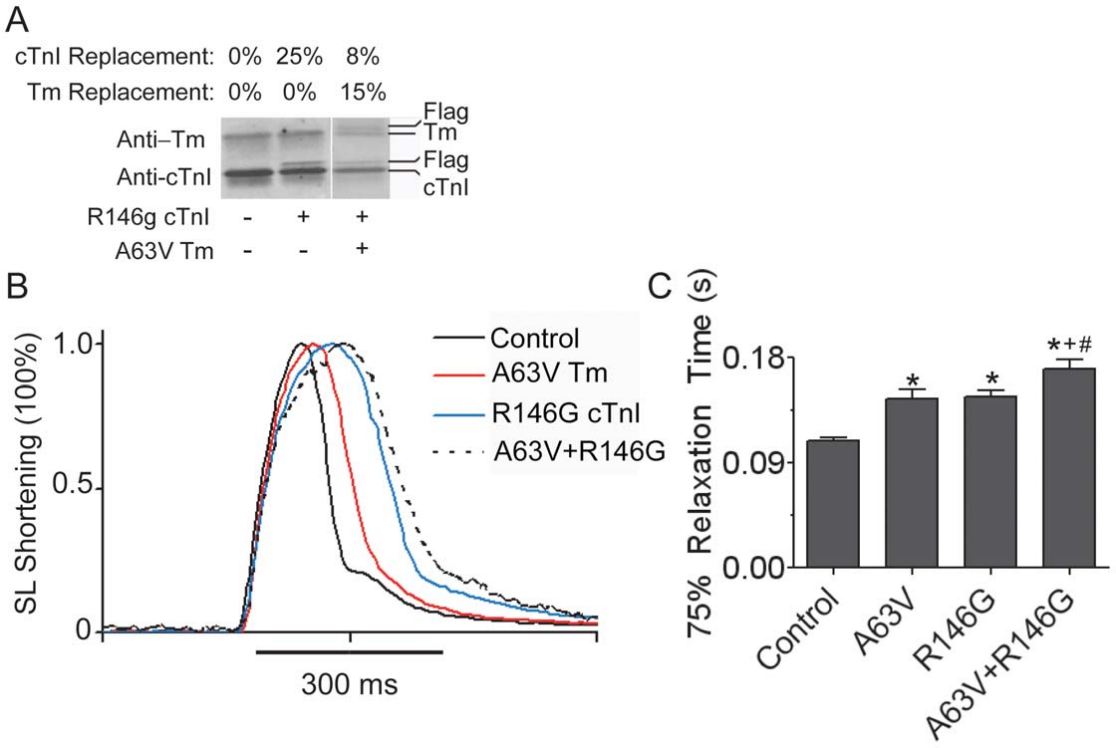
Intact single myocyte shortening from double activating mutations in αTm and cTnI. (**A**) Representative Western blot showing non-transduced (WT), R146G cTnI, and A63V Tm + R146G cTnI myocytes. Blots were probed with anti-Tm and anti-cTnI antibodies. (**B**) Sarcomere shortening transients from WT, A63V Tm, R146G cTnI, and A63V+R146G transduced myocytes. Traces were normalized to peak shortening to emphasize the mutant dependent slowing of relaxation. (**C**) Summary of 75% sarcomere relaxation time. Relaxation time was determined by calculating the difference from the time of peak shortening to 75% relaxation. Values are represented as the mean+SEM and Newman-Keuls post hoc comparisons defined as follows: (*) different from WT, (+) different from R146G cTnI, (#) different from A63V Tm, P<0.05, myocytes were derived from a minimum of 5 different rat heart isolations with n = 45 WT, n = 40 R146G, and n = 40 A63V+R146G myocytes.

We tested three different CM mutant alleles shown previously and confirmed here to cause weak, moderate, or strong myofilament activation when tested individually (Table 1). The primary functional effects of these CM mutants, when expressed together at the working myocyte level, are presently unknown. We first tested the functional effects of co-expressing a moderate myofilament activator, αTmA63V, with a weak activator, cTnIR146G. Unloaded sarcomere shortening in intact adult rat cardiac myocytes at physiologic temperatures (37°C) was examined four days after dual adenoviral gene transfer. A representative Western blot in Figure 2A demonstrates the targeted stoichiometric replacement of αTm and cTnI achieved with dual gene transfer of TmA63V and cTnI R146G. In this pairing, A63V has 15±7% replacement of native αTm, and cTnIR146G achieves approximately 20±5% replacement of native cTnI. This level of replacement is less than previous reports of single gene transfer with these particular mutants [31], [32], [45]. Representative raw sarcomere shortening traces normalized to peak shortening amplitude illustrate the pronounced slowing of relaxation characteristic of single TmA63V and cTnIR146G mutant myocytes (Figure 2B). Dual gene transfer of these mutants (TmA63V + cTnIR146G) had an additive effect when compared to either mutant alone in which 75% relaxation time was significantly slowed by 30% (Figure 2C).

**Table 1:** Myocyte shortening and Ca^2+^ transient parameters.

Sarcomere length (SL) shortening measurements from single intact cardiac myocytes						
	Baseline SL (mm)	Amplitude (nm)	*Dep vel* (%)	50% RTP (ms)	75% RTP (ms)	90% RTP (ms)
WT (n = 63)	1.81±0.01	149±13	17±1	90±5	152±7	334±27
R193H cTnI (n = 64)	1.71±0.01*^,#^	172±11*	20±1	198±15*^,#^	355±27*^,#^	646±42*^,#^
A63V Tm (n = 29)	1.80±0.01^+^	189±22	16±2	141±14*^,+^	246±22*^,+^	464±43*^,+^
A63V+R193H (n = 29)	1.71±0.02*^,#^	166±16	18±1	250±27*^,+,#^	493±60*^,+,#^	802±77*^,+,#^
G159D cTnC (n = 36)	1.85±0.01*^,+^	166±14	20±1	72±41^+^	131±9^+^	283±24^+,#^
G159D+R193H (n = 36)	1.75±0.02*^,+,#^	175±14	21±1	140±8*^,+,#^	280±16*^,+,#^	557±33*^,+,#^

Calcium transient measurements from single intact cardiac myocytes						
	Baseline (360/380)	Amplitude (%BL)	*D vel Ca^2+^* (%)	50% DTP (ms)	75% DTP (ms)	90% DTP (ms)
WT (n = 43)	0.80±0.01	18.1±1.6	82±3	204±15	345±27	532±36
R193H cTnI (n = 44)	0.79±0.01	21.8±1.5^#^	79±12	285±13*	460±24*	729±42*
A63V Tm (n = 19)	0.78±0.01	27.9±2.5*^,+^	77±7	278±14*	464±25*^,+^	757±47*
A63V+R193H (n = 29)	0.67±0.01*	14.5±2.2^+,#^	75±9	362±22*^,+,#^	639±41*^,+,#^	1,090±70*^,+,#^
G159D cTnC (n = 19)	0.79±0.02	14.3±2.2^+^	74±8	195±15^+^	343±25^+^	551±34^+^
G159D+R193H (n = 26)	0.88±0.02	16.0±1.9	86±4	268±10*^,#^	430±17*^,#^	656±30^+^

Wild type (WT) is the control group and consists of combined data from non-transduced, AdcTnI (WT), and AdcTnIFlag. A t-test indicated these data were not significantly different (P>0.32). Mechanical and Ca^2+^ transient data from each experimental and control group were collected simultaneous, thus the WT data represents the control group for each pairing. *1X4-way ANOVA* was used to statistically compare dual gene transfer pairings to single gene transfer and control groups. Values are represented as the mean ± SEM and Newman-Keuls post hoc comparisons defined as follows: (*) different from WT, (+) different from R193H cTnI, (#) different from A63V Tm or G159D cTnC, P<0.05, n = 19–64 myocytes derived from 5 different rat heart isolations. Shortening velocity is normalized to the peak shortening amplitude (Amp). Relaxation time is calculated from peak shortening to 50, 75, or 90% of relaxation. Key: BL =  baseline, Dep Vel  =  sarcomere length shortening velocity, RTP  =  relaxation time from peak, D Vel Ca^2+^  =  rate of rise of the Ca^2+^ transient, and DTP  =  Ca^2+^ transient decay time from peak.

**Figure 3:**
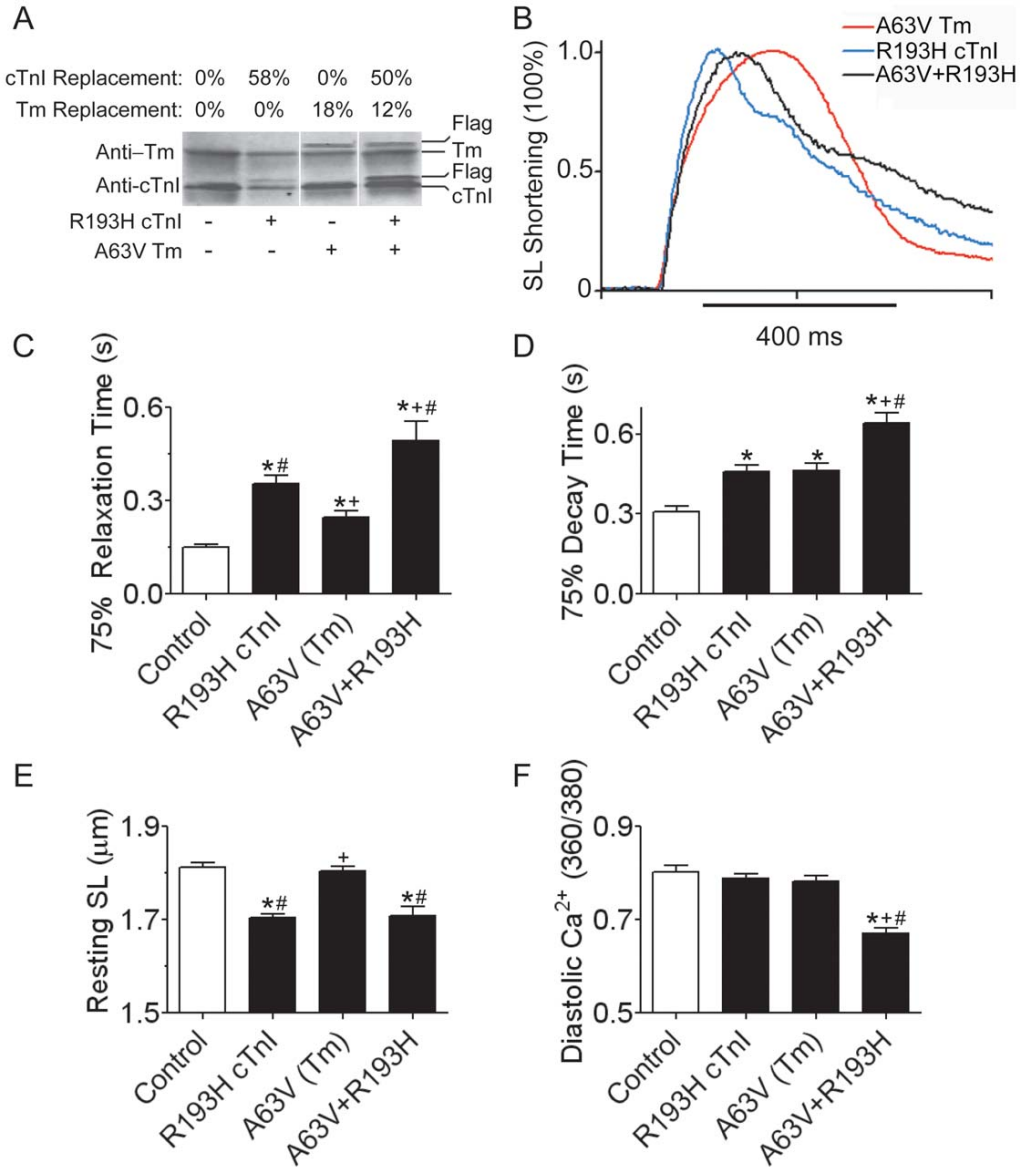
Intact single myocyte shortening and Ca^2+^ transients from double activating αTm and RCM cTnI mutants. (**A**) Representative Western blot from myocytes transduced with R193H cTnI, A63V Tm, or both R193H cTnI and A63V Tm. Non-transduced myocytes served as controls. Blots were probed with anti-Tm, anti-cTnI antibodies and anti-TnC was used as a loading control (*not shown*). (**B**) Representative sarcomere shortening transients from A63V Tm (HCM), R193H cTnI (RCM), and A63V+R193H transduced myocytes. Traces were normalized to peak shortening to emphasize the mutant dependent slowing of relaxation. (**C**) Summary of 75% sarcomere relaxation time and (**D**) Ca^2+^ transient decay time. Relaxation and decay times were determined by calculating the difference from the time of peak shortening/fluorescence to 75% relaxation/decay. (**E**) Summary of baseline sarcomere lengths and (**F**) resting Ca^2+^ for all of the experimental groups. Values are represented as the mean + SEM, and Newman-Keuls post hoc comparisons defined as follows: (*) different from WT, (+) different from R193H cTnI, (#) different from A63V Tm or G159D cTnC, P<0.05, myocytes were derived from a minimum of 5 different rat heart isolations with n = 63/43 WT, n = 64/44 R193H, n = 29/19 A63V,and n = 29/29 A63V+R193H myocytes used for shortening/Ca^2+^ cycling experiments.

**Figure 4:**
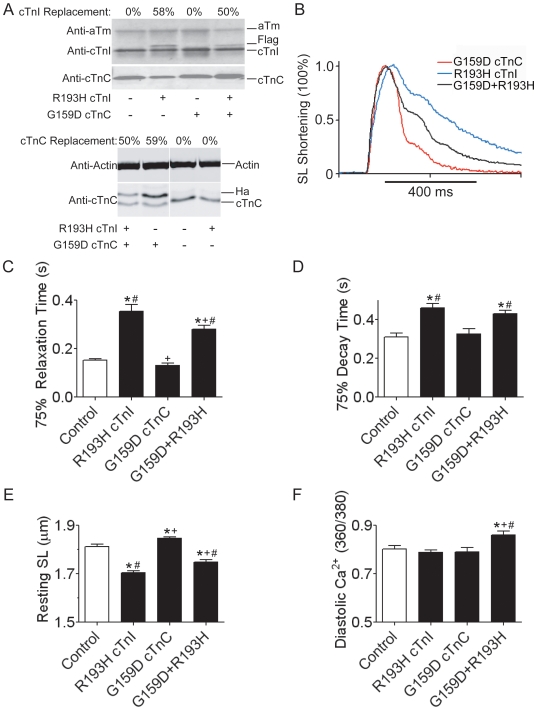
Intact single myocyte shortening and Ca^2+^ transients from a deactivating cTnC and activating cTnI mutant combination. (**A**) Representative Western blot from myocytes transduced with R193H cTnI, G159D cTnC, or both R193H cTnI and G159D cTnC. Non-transduced myocytes served as controls. Blots were probed with anti-cTnI (top panel) and anti-cTnC (bottom panel) antibodies and anti-Tm was used as a loading control. (**B**) Representative sarcomere shortening transients from G159D cTnC, R193H cTnI, and G159D cTnC + R193H cTnI transduced myocytes. Traces were normalized to peak shortening to emphasize the mutant dependent slowing of relaxation. (**C**) Summary of 75% sarcomere relaxation time and (**D**) Ca^2+^ transient decay time. Relaxation and decay times were determined by calculating the difference from the time of peak shortening/fluorescence to 75% relaxation/decay. (**E**) Summary of baseline sarcomere lengths and (**F**) resting Ca^2+^ for all of the experimental groups. Values are represented as the mean + SEM, and Newman-Keuls post hoc comparsions defined as follows: (*) different from WT, (+) different from R193H cTnI, (#) different from G159D cTnC, P<0.05, myocytes were derived from a minimum of 5 different rat heart isolations with n = 63/43 WT, n = 64/44 R193H, n = 36/19 G159D,and n = 36/26 G159D+R193H myocytes used for shortening/Ca^2+^ cycling experiments.

The following set of experiments tested the combination of a strong myofilament activator, cTnIR193H, with a moderate activator, TmA63V, on myocyte function. Figure 3A is a representative Western blot probed with anti-Tm and anti-cTnI antibodies showing the targeted stoichiometric replacement of cTnIR193H, TmA63V, and A63V+R193H (dual gene transfer group) relative to WT myocytes (Figure 3A) three days after gene transfer. With single gene transfer, TmA63V achieved 18±5% replacement and cTnIR193H achieved 58±3%, and with dual gene transfer the replacement of both mutant proteins was reduced by 6–8% (Figure 3A). In unloaded functional assays representative sarcomere shortening transients illustrate the effects of αTmA63V, cTnIR193H, and A63V+R193H on cardiac myocyte relaxation (Figure 3B). In this assay direct comparisons of myocyte shortening demonstrated that R193H myocytes are slower to relax than A63V mutants at 50, 75, and 90% relaxation times (Table 1). Dual gene transfer of TmA63V and cTnIR193H produced an additive slowing of relaxation time that was 40% greater than that of R193H alone (Figure 3C). Ca^2+^ transients were simultaneously measured yielding results similar to mechanical relaxation (Table 1, Figure 3D). Single gene transfer of A63V and R193H slowed Ca^2+^ transient decay time to similar extents, relative to WT myocytes (Figure 4C, Table 1); whereas, co-expression of TmA63V and cTnIR193H slowed Ca^2+^ transient decay by 39% relative to cTnIR193H alone (Figure 3C, Table 1). These data show that within the context of the physiologically intact unloaded myocyte, the dual incorporation of two myofilament activators from different sarcomeric loci directly and additively increase the magnitude of cellular diastolic dysfunction beyond the phenotype of the strongest activating allele within the pairing. A comparison of results obtained from the moderate and weak activator pairing (A63V+R146G) versus the moderate and strong activator pairing (A63V+R193H) also underscores the relationship between the nature of the activators (weak, moderate, or strong) and the resulting degree of diastolic dysfunction, which worsens in combinations with stronger activating alleles like the A63V+R193H.

Given the role of gene dosage as a critical factor in determining diseased phenotypes both *in vivo* [8]–[10], [15], [22], [35], [38] and *in vitro* [22], [50], [51] it is possible that the additive outcomes in this pairing are due to the global increase in mutant sarcomeric protein incorporation. While a gene dosage hypothesis was not directly tested in this study, we propose that the basis for additive outcomes in A63V+R193H mutant myocytes cannot be entirely accounted for by an increased amount of total mutant gene dosage, as the total amount of mutant incorporation was ∼60% for both A63V+R193H and R193H alone (Figure 3A). Despite similar amounts of CM mutant filaments incorporating into the sarcomere, the A63V+R193H pairing still additively slowed relaxation beyond that of R193H cTnI.

As recently shown, the incorporation of cTnIR193H into the intact sarcomere acutely remodels cardiac myocytes to shortened resting sarcomere lengths independent of diastolic [Ca^2+^] [22]. TmA63V had no effects on resting sarcomere lengths or diastolic Ca^2+^ (Figure 3E and F). Myocytes with A63V+R193H had a phenotype similar to R193H myocytes, as A63V+R193H myocytes also transitioned to short resting sarcomere lengths independent of elevated diastolic Ca^2+^ (Figure 3E and F). This suggests that cTnIR193H has a dominant effect over TmA63V to shortening resting sarcomere lengths in intact cardiac myocytes. Additional contractile parameters, including sarcomere shortening amplitude, were not different between groups (Table 1). A63V mutant myocytes, however, had a significant increase in Ca^2+^ transient amplitude relative to WT and dual gene transfer groups (Table 1). When TmA63V was coexpressed with cTnIR193H, Ca^2+^ transient amplitude was not different from WT myocytes (Table 1) highlighting the unpredicted combinatorial effects of two mutant thin filament proteins on Ca^2+^ cycling.

### Myofilament CM Activator and Deactivator Mutants in Combination

**Figure 5:**
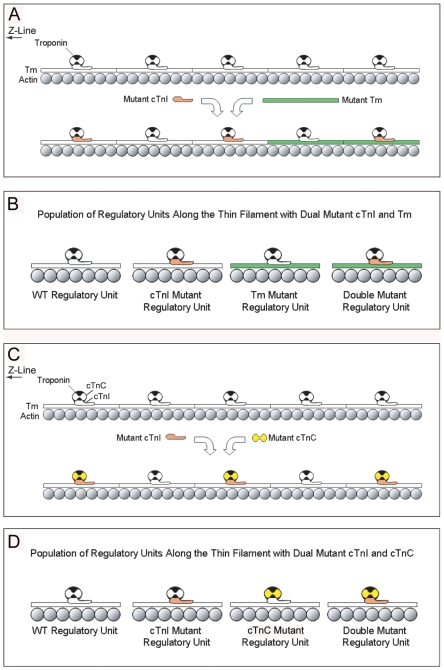
Model of dual gene transfer incorporation of mutant Tm + cTnI and cTnC + cTnI combinations. (**A**) Native cTnI and αTm is shown in white. This model predicts that mutant Tm (green) has ordered incorporation starting at the pointed end and moving towards the Z-line, while the simultaneous transduction of mutant cTnI (red) will stochastically incorporate along the thin filament. As such double mutant regulatory units containing both mutant Tm and cTnI are located at the pointed end of the thin filament in this dual gene transfer approach. (**B**) Schematic depicting the predicted population of regulatory units that decorate the thin filament in this model system and in CM patients. Four populations of regulatory units consisting of wild type (WT), mutant cTnI alone, mutant Tm alone, and both mutant cTnI and TM (double mutant) are predicted to constitute the thin filament regulatory system as a whole. The relative proportion of units will be directly related to the percent replacement of native Tm and cTnI that in a patient will reach an even and random distribution overtime as the thin filaments turn-over. In our model system, TmA63V + cTnIR193H myocytes very few double mutant regulatory units are predicted as R193H mutant cTnI will be randomly distributed while only 10% should contain A63V Tm. (**C**) Native cTnI and cTnC are shown in white. This model predicts that like mutant cTnI (red), mutant cTnC (yellow) will have stochastic incorporation. Regulatory units containing both mutant cTnC and cTnI are randomly decorated along the entire length of the thin filament. The incorporation of mutant cTnC is predicted to follow cTnI. (**D**) Schematic depicting the predicted population of regulatory units that decorate the thin filament with this dual gene transfer approach. Four populations consisting of wild type (WT), mutant cTnI alone, mutant cTnC alone, and both mutant cTnI and cTnC (double mutant) regulatory units are predicted to constitute the thin filament regulatory system in our model and in CM patients. If mutant cTnC follows cTnI then the population of single mutant regulatory units should approach zero with double mutant and WT regulatory units being more prominent and in a 50∶50 distribution. The relative proportion of double mutant regulatory units is predicted to be directly related to the percent replacement of native cTnC and cTnI.

CM sarcomeric proteins can directly cause thin filament deactivation [52]–[55]. It is therefore possible that individuals could inherit one CM mutant myofilament activator allele and one CM mutant deactivator allele. The functional outcomes of such a pairing have not been addressed in any model system to our knowledge. Thus, co-transduction of intact adult cardiac myocytes with CM myofilament deactivator cTnCG159D together with myofilament activator cTnIR193H was examined. Figure 4A is a representative Western blot probed with anti-Tm, anti-cTnI, and anti-cTnC antibodies showing the targeted stoichiometric replacement of native cTnI with R193H cTnI. R193H cTnI replacement reached 58±5% with single gene transfer and was slightly reduced to 50±2% when co-expressed with cTnCG159D (Figure 5A, top panel). The separation between native cTnC and HA-tagged cTnCG159D was resolved on an 18% SDS-page gel as shown in the bottom panel of Figure 5A. G159D cTnC replacement reached 60±4% with single gene transfer and was slightly reduced to 50±2% when co-expressed with cTnIR193H. Representative sarcomere shortening traces (Figure 4B) demonstrate the 2.5 fold difference in myocyte relaxation between cTnCG159D and cTnIR193H mutant myocytes. R193H cTnI significantly slowed relaxation relative to cTnCG159D and myocytes expressing native cTnC/cTnI (Figure 4C and Table 1). Dual gene transfer of cTnCG159D and cTnIR193H resulted in a hybrid phenotype (Figure 4C), in which the 75% relaxation time was 21% faster in G159D+R193H myocytes relative to R193H myocytes (Figure 4C); however, the G159D+R193H myocytes were still slower to relax when compared to cTnCG159D alone and WT myocytes (Figure 4C). The decay time of the Ca^2+^ transient was also decreased in G159D+R193H myocyte (Figure 4D), but this change was not statistically different from cTnIR193H myocyte Ca^2+^ decay times.

The cTnIR193H mutant alone exhibited a characteristic Ca^2+^-independent transition to shortened resting sarcomere lengths (Figure 4E and F) [22]. The cTnCG159D CM mutant alone had the opposite effect such that resting sarcomere lengths were significantly longer than both WT and R193H myocytes, in addition to having elevated resting diastolic Ca^2+^ (Figure 4E and F). Baseline sarcomere lengths in G159D+R193H myocytes were significantly longer than those measured in R193H myocytes, but still significantly shorter than both WT and G159D. The partial correction of R193H-dependent short resting sarcomere lengths by cTnCG159D provides further evidence that these mutants can combine to blunt prominent phenotypic characteristics of a given CM mutant allele. The strong activator cTnIR193H still exerted a dominant effect over the deactivator cTnCG159D to slow relaxation and shorten resting sarcomere length. Collectively, these data demonstrate that within the context of the physiologically intact unloaded myocyte coexpressing a deactivating and an activating sarcomeric allele from different loci partially mitigates the primary effects elicited by each mutant alone.

## Discussion

These results demonstrate that double cardiomyopathy mutants, with mild to strong myofilament activating properties combine to worsen cellular diastolic dysfunction. These additive effects on mechanical performance (slowed relaxation and Ca^2+^ decay) show evidence of a functional ceiling for some mutant combinations suggesting there are limits to mechanical dysfunction induced by compound mutations. We also studied in combination CM mutants with distinct activating and deactivating properties. *A priori*, such combination could result in a dominant effect or, alternatively, a hybrid functional outcome. Findings here show that combining CM mutants with opposing effects on myofilament function (activator + deactivator) produces a hybrid outcome and evidence of functional neutralization at the intact myocyte level. This finding raises the possibility that two CM mutant alleles, that in isolation markedly alter cell performance to cause CM, could in combination yield near normal cellular physiology. These data are used to develop a working model (detailed below) for predicting molecular outcomes on the >100,000 possible CM double mutant combinations.

### Double Activating Alleles: Evidence for Additive and Functional Threshold Effects

The cause and effect relationship for CM patients with multiple disease alleles is poorly understood due to a myriad of compounding factors including environment, genetic modifiers, increased mutant allele dosage, or allele-specific functional interactions that likely contribute to the severity of the disease presentation [4], [9], [56], [57]. Here, two different combinations of double activating alleles were studied: weak + moderate (TmA63V+ cTnIR146G) and a moderate + strong (TmA63V+cTnIR193H). Combinatorial analysis showed additive effects to further slow relaxation and Ca^2+^ transient decay beyond either mutant studied in isolation. This worsening of cellular diastolic dysfunction provides a biophysical basis for the increased disease severity in case reports of double heterozygous CM patients. The additive slowing of mechanical relaxation in double mutant myocytes underscores the contribution of both diseased alleles to the cellular phenotype especially as the total amount of mutant incorporation was similar between the double mutants when compared to single mutant myocytes (Figure 3A). It is well known that myocyte contraction, and by association overall organ physiology, is highly dependent on the thin filament regulatory proteins functioning together as a cooperative unit [39]. In the three-state model of thin filament regulation two activating ligands Ca^2+^ and strong cross-bridge attachment cause the regulatory proteins to dynamically transition between blocked, closed and open positions thereby orchestrating the cyclical bouts of diastole and systole in the beating heart. The transitions between these regulatory states are initiated by Ca^2+^ binding to TnC, which switches TnI from an inhibitory to a permissive position thereby permitting Tm movement and access for strong force generating cross-bridges. The ability of these CM activating alleles to combine properties is at least in part due to the distinct and vital roles described above that each gene product (cTnI, cTnC, and Tm) plays in thin filament regulation.

In this study we ascribe the worsening of myocyte mechanical performance to the combined effects that gain of function mutations in cTnI and Tm have on enhancing Ca^2+^-mediated thin filament activation [22], [38], [40], [58]. Individual mutations in cTnI's C-teminus (e.g. R146G and R193H) alter TnI switch function which in turn heightens myofilament Ca^2+^ sensitivity and thus sarcomere relaxation [59], [60]. Whereas, gain of function mutations in Tm increase Tm flexibility lowering the energy required for Tm movement and making the thin filament more readily activated [31], [61]. Coexpressing these activating mutant alleles in cardiac myocytes coupled their altered biophysical properties to further heighten activation at even lower Ca^2+^ concentrations when compared to their single mutant counterparts. This heightened activation manifests itself as a more severe cellular diastolic dysfunction, as the sarcomere remains contracted with decaying Ca^2+^ concentrations. While the A63V+R193H double activator combination slowed relaxation beyond that of either mutation studied in isolation, the net effect was not precisely additive on a one to one basis as the sum total of relaxation times for single R193H and A63V myocytes (355+246 = 601 ms, 75% Relaxation) was ∼17% greater than those measured in A63V+R193H dual gene transfer myocytes (493 ms). This suggests that the A63V+R193H combination may reach a functional threshold or a physiologic limit in which additional mutant incorporation no longer alters thin filament activation similar to previous reports where extracting only a fraction of troponin complexes (as little as 5%) results in nearly maximal Ca^2+^ independent thin filament activation [62]. These results support the prominent role of long range and nearest neighbor interactions by Tn and Tm and a possible saturation point at which further thin filament modification would not alter Ca^2+^ activation beyond this saturation point. The functional threshold attained by the A63V+R193H myocytes in this case could be ascribed to the lower gene dosage of both mutants attained in the dual gene transfer model. Perhaps with increased A63V Tm incorporation the alterations in diastolic dysfunction could be precisely additive. Alternatively, A63V mutant Tm and R193H mutant cTnI may overlap in terms of their biophysical roles in thin filament regulation and thus only combine to fractionally worsen diastolic dysfunction leaving open the question of possible compound CM mutant combinations that may encroach on a physiologic limit where the thin filament attains complete Ca^2+^-independent dis-inhibition and maximal tension generation during relaxation conditions. The R193H cTnI mutant alone encroaches on this limit as it has a dominant effect in all combinations to cause a Ca^2+^ independent diastolic tone [22], [30].

An equally important factor that likely impacts the additive effects of two activating mutant alleles is the combined incorporation of each mutant into the sarcomere given the central role thin filament stoichiometry and cooperativity plays in the regulation of contraction [40]. The thin filament regulatory system is comprised of 24 multiplexed regulatory units that span the 1 um length of actin with a single regulatory unit (Tm, cTnC, cTnT, and cTnI) for every 7 actin monomers [40]. Each regulatory unit is coupled by Tm permitting neighboring and long range interactions between other regulatory units [40], [63]–[65]. Studies have shown that Tm incorporates from the pointed end to the Z-line of the sarcomere, while cTnI incorporation is stochastic and uniformly distributed along actin [47]. The differential modes of incorporation have implications for the double mutants studied here and for double heterozygous patients. The percentage of CM mutant protein expression in patients is still unknown but expected at around 50% per mutant allele from different genetic loci. Given the estimated total of CM mutant allele expression and the modes of thin filament incorporation, we postulate that double heterozygous genotypes produce a mixed population of regulatory units (Figure 5A) in which a proportion of them are WT (unaffected), mutant cTnI only, mutant Tm only, and mutant cTnI+Tm mixed regulatory units (Figure 5B). Over a patient's lifetime the myofilaments will continually turn-over as the half-life of thin filament proteins is 3–5.5 days [47], [66]. As a result the location of these mixed regulatory units along the thin filament will constantly change likely yielding an even and random distribution of mixed, single mutant, and WT regulatory units in a CM heart despite the ordered incorporation of Tm in an acute setting. At present the effects of double mutant regulatory units alone on regulatory function remains unknown. In our A63V+R193H experiment, the low percentage of TmA63V incorporation demonstrates that the change in myocyte physiology is attributed to very few double mutant regulatory units in tandem with single mutant regulatory units (Figure 5A and B) as only a small percentage of regulatory units contain mutant Tm. Despite this small number, the additive outcomes suggests that nearest neighbor and long range cooperative interactions between single mutant regulatory units are more responsible for the enhanced activation in the A63V+R193H pairing.

### Neutralization Effects of Combined Activating and Deactivating Alleles

A critical insight garnered from this study is that two CM mutant alleles in combination may not necessarily result in a more severe cellular dysfunction. This is evident from the deactivator + activator combination (cTnIR193H + cTnCG159D) in which the diastolic dysfunction characteristic of single cTnIR193H mutant was neutralized in part by cTnCG159D producing an intermediate activating phenotype. The G159D mutation appears to play a structural role in the troponin complex, but also interacts with cTnI's N-terminus [41], [54], [67], [68]. Chimeric studies showed that cTnI's N-terminus influences myofilament Ca^2+^ sensitivity [69]; therefore, cTnC G159D may effect hastening of relaxation through this interaction with the N-terminus of cTnI. In this case, the complimentary but opposing biophysical properties of cTnC G159D and cTnI R193H appear to counteract each other. Thus, at the organ level co-expressing an activating and deactivating mutant allele could produce a less severe phenotype than either allele acting alone.

In contrast to the mutant Tm and cTnI pairings, the modification of the thin filament with cTnCG159D and cTnIR193H is modeled with a different distribution pattern and higher percentage of double mutant regulatory units (Figure 5C and D). It is currently unknown if cTnC has ordered or stochastic incorporation into the sarcomere. Because sub-cellular pools of new cTnC have not been discovered [66], cTnC is predicted to stochastically incorporate into the sarcomere similar to cTnI but with a delayed time course due to the longer half-life of cTnC (5 days versus 3 days, [47], [66]). In this study there was 50% incorporation of cTnCG159D and cTnIR193H indicating that in mutant cTnC and cTnI combinations at least half of the regulatory units should contain a mutant cTnC or cTnI (Figure 5C and D). By chance alone there is a higher probability that both mutants will incorporate into the same regulatory unit than the number predicted for Tm + cTnI pairings (Figure 5A) in this model system. If cTnC incorporation follows the cTnI pattern then the affected regulatory units (∼1/2) should contain both mutant proteins (Figure 5C and D) with the rest of the regulatory units being WT. In a double heterozygous CM patient with mutant troponin subunits, the distribution of the mixed regulatory units should achieve a random and even distribution across the thin filament similar to the equilibrium achieved with Tm+TnI combinations. If cTnC follows cTnI replacement patients with this mutant allele combination are predicted to have a 50∶50 distribution of double mutant and WT regulatory units. In this case the mutant regulatory units exert a dominant effect over WT.

### Predictive Model for Compound CM Mutants

**Figure 6:**
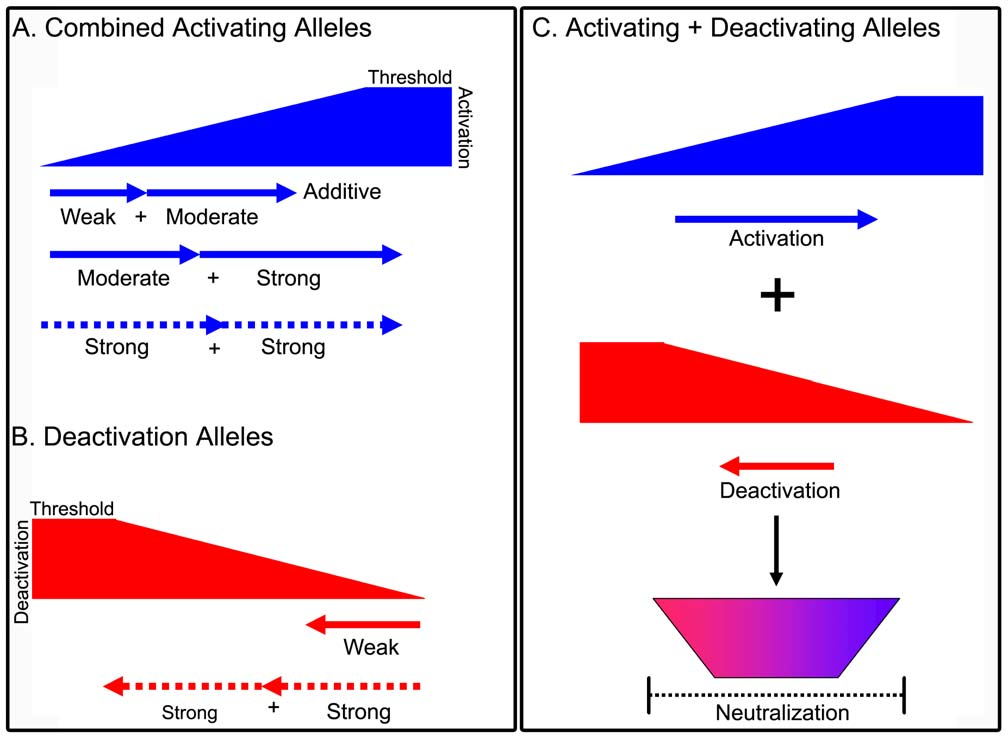
Working model of additive and neutralizing outcomes for double heterozygous genotypes based on the combine effects of allele-specific thin filament activation. In the models depicted in all panels the arrows represent the individual allele as a linear vector. The length of the arrow represent the magnitude of activation/deactivation of the thin filament caused by the mutant allele alone, which is classified as weak, moderate, or strong. The arrow's direction represents the allele's alteration of thin filament activation in which a rightward pointing arrow (positive direction) represents activation and a leftward pointing (negative direction) arrow represents deactivation. (A) Double Activating Allele Model: the positive sloping gradient represents the additive slowing of relaxation and heightened severity of diastolic dysfunction that occurs with the combination of two activating mutant alleles from different sarcomeric loci. The combination of two activating alleles is represented by adding two activating allele vectors together, which yields a higher degree of activation and thus more severe diastolic dysfunction. Some double activating allele combinations may cause the thin filament to become saturated thereby approaching a threshold of activation in which the combination of two vectors is not additive on a one to one basis. (B) Double Deactivating Allele Model: the negative sloping gradient represents the predicted additive consequence to accelerate relaxation and reduce contractility that occurs with the combination of two deactivating mutant alleles from different sarcomeric loci. The combination of two deactivating alleles is represented by adding two activating allele vectors together but in the negative direction, which yields a higher degree of deactivation and thus altered contractility with enhanced relaxation. Some double deactivating allele combinations may also result in saturation thereby approaching a threshold of deactivation. (C) Mixed Model of Activating + Deactivating Alleles: the combination of two alleles with different activation properties is depicted by the addition of a positive gradient and vector (activating allele) and a negative gradient and vector (deactivating allele). This combination results in mitigating effects, in which the primary phenotypes of each individual mutant are neutralized (represented by the trapezoid) when the opposing alleles are co-expressed.

Using experimental data from activating and deactivating sarcomeric CM mutants obtained here and elsewhere as foundations, a working model was developed to promote predictive insights into myocyte function of the theoretically possible tens of thousands of compound CM combinations. The model was constructed by categorizing single mutant CM alleles by their functional properties as myofilament activators or deactivators, and by the strength with which these properties have been altered. The activation properties of numerous single sarcomeric mutants in both the thick and thin filament have already been characterized [22], [29], [30], [36], [37]. We propose all CM sarcomeric can be incorporated into the model as they all fundamentally effect myofilament regulation [8], [29], [36], [37], [70]–[73]. The model employs vector addition to illustrate the merging of the primary functional effects of each distinct mutant allele in a given double heterozygous combination. Here a linear vector represents a single mutant allele (Figure 6). The length of the vector reflects the individual strength of activation/deactivation (weak, moderate, or strong; Figure 6), and the direction of the vector represents the activation properties of a mutant (right arrow  =  activator, left arrow  =  deactivation; Figure 6A and B). The single mutant vectors of a double heterozygous combination are then added head to tail. Two vectors (alleles) in the same direction are summated to give the net outcome. If the net direction points towards activation results in more severe diastolic dysfunction as represented by the blue positive sloping gradient (Figure 6A). By contrast, summated myofilament deactivators are predicted to hasten relaxation and alter contractility [52], [55], [74] as represented by the red negative sloping gradient (Figure 6A). Support for this model comes from the data presented here in which a weak/strong and a moderate/strong activator combination both additively worsened diastolic function. This model is further supported by the heightened pathology and increased mortality reported in a CM double heterozygous mouse model that expresses both strong (R403Q Myosin) and moderate activator (G203S cTnI) alleles [28]. The model incorporates functional ceiling effects obtained by combining two strong activator alleles (or two strong deactivating alleles) where the myofilament activation (or deactivaiton) reaches a functional threshold and is no longer additive on a one to one basis.

This model also incorporates the neutralizing effects of activator and deactivator alleles in combination (Figure 6C). Here, the deactivator and the activator are combined to predict the outcome. The degree of neutralization in activator/deactivator combinations is represented by the trapezoid in which the spectrum of colors (red to purple to blue) depicts the addition of two opposing activating alleles of different strengths. Support for neutralizing effects comes directly from activator/deactivator alleles in combination (Figure 6), as well as from a mouse model coexpressing a CM-linked activating and deactivating Tm [75]. Further support for this model comes from a recent study demonstrating that the expression of moderate and mild deactivating cTnC mutants can elicit significant reductions in myocyte contractility [52]. An important outcome of the experimental results and model is that offspring inheriting two malignant CM alleles, that separately cause myofilament activation in one parent and deactivation in the other, could combine to produce a functional neutralization and benign presentation.

For experimental purposes we did not employ the more prevalent double heterozygous genotypes, which predominantly consist of MyHC and MyBP-C mutant allele combinations. As myosin is a vital ligand required for maximal activation of tension development [42], [44], β-MHC mutant alleles can be classified within this activator/deactivator paradigm and thus are well suited for our working model. The R403Q MHC can be considered a moderate-strong activating allele [8], [71]–[73], and based on our model, the functional outcome of having an R403Q β-MHC allele paired with another activator would be predicted to cause an additive worsening of diastolic dysfunction (Figure 6). While this activating activating/deactivating paradigm should be tested on other mutant sarcomeric alleles, support for our model can be derived from double heterozygous patients with a combined myosin and troponin mutation that have early morbidity and an increased incidence of surgical treatment and sudden cardiac death, as well as the increased mortality in the MyHC R403Q + G203S cTnI double heterozygous mice described above [76].

In conclusion, our findings show at the cellular/molecular level double mutant thin filament proteins have direct and combinatorial effects to alter cellular diastolic function. The mechanistic basis for additive/mitigating effects cannot be solely due to total mutant gene dosage alone, but also appears to be highly dependent on the population and location of double mutant regulatory units along the thin filament and the biophysical properties of the effected sarcomeric protein in terms of the activating/deactivating nature and the strength of these mutant alleles. These results also underscore that the primary outcome for double heterozygous patients may not necessarily cause a worsening of the disease phenotype particularly in cases where the genotype produces an activating and a deactivating mutant myofilament protein.
